# Eco-Friendly Synthesis of Chitosan–Fatty Acid Nano Micelles and Their Differential Antibacterial Activity Against *Escherichia coli* and *Bacillus subtilis*

**DOI:** 10.3390/jfb16100373

**Published:** 2025-10-07

**Authors:** Alfio Pulvirenti, Valentina Verdoliva, Viviana De Luca, Serena Traboni, Clemente Capasso, Stefania De Luca

**Affiliations:** 1 Department of Biomedical Sciences, Institute of Biostructures and Bioimaging, National Research Council (CNR), Via P. Castellino, 111, 80131 Naples, Italy; alfio.pulvirenti86@gmail.com; 2 Department of Environmental, Biological and Pharmaceutical Sciences and Technologies, Institute of Crystallography, National Research Council (CNR), Via Vivaldi, 43, 81100 Caserta, Italy; valentina.verdoliva@cnr.it; 3 Department of Biology, Agriculture and Food Sciences, Institute of Biosciences and Bioresources, National Research Council (CNR), Via P. Castellino, 111, 80131 Naples, Italy; viviana.deluca@ibbr.cnr.it (V.D.L.); clemente.capasso@cnr.it (C.C.); 4 Department of Chemical Sciences, University of Naples Federico II, Strada Comunale Cinthia 26, 80126 Naples, Italy; serena.traboni@unina.it

**Keywords:** chitosan-fatty acid conjugates, eco-friendly protocol, chitosan-based nanoparticles, fatty acid antimicrobial activity, chitosan enhanced solubility

## Abstract

Chitosan-based nanoparticles were prepared using an eco-friendly chemical procedure that conjugates natural fatty acids to the backbone of chitosan. This consists of reacting two molecules in the absence of a solvent and using microwaves to promote the chemical transformation. Both conditions make the whole chemical process more eco-compatible in terms of reagents and energy consumption. The chemical structure and the self-association behavior of chitosan–fatty acid conjugates were characterized by FT-IR, NMR, and dynamic light scattering. The conjugates displayed an enhanced solubility and efficient self-assembly in aqueous solution. The antimicrobial activity of the resulting nanoparticles was evaluated against *Escherichia coli* (Gram-negative) and *Bacillus subtilis* (Gram-positive). The micelles significantly inhibited *E. coli* growth (35–60%), even at relatively low concentrations, whereas negligible activity was observed against *B. subtilis*. The antibacterial efficacy appears to arise primarily from the ability of the developed nanostructured conjugates to perturb bacterial membranes. These results support the potential of chitosan–fatty acid conjugates as sustainable nanomaterials for biomedical applications, particularly as eco-friendly antimicrobial agents. Future work will evaluate their activity against other Gram-positive pathogens and explore their use in drug delivery.

## 1. Introduction

Due to the widespread emergence of new strains of antibiotic-resistant pathogens, the need to discover and develop new antimicrobial systems has become increasingly urgent. To address this public health issue, several solutions have been proposed, including the use of nanoparticles with antibacterial properties, surfactants, antimicrobial peptides, and antibacterial lipids, such as natural fatty acids [1].

Fatty acids (FAs), naturally occurring compounds, exhibit a broad spectrum of biological activities and, among them, possess well-known antimicrobial properties [2]. In this regard, the FA potential has remained essentially underexploited, largely due to their lack of solubility in aqueous media along with the widespread use of antibiotics in healthcare over the past 80 years. The primary antibacterial action of FAs arises from the insertion of their hydrophobic alkyl chains into the bacterial lipid membrane, leading to membrane disruption, cell lysis, and ultimately bacterial death. This mechanism was proven to be also mediated by membrane electrostatic interactions or membrane surface accumulation by which the alkyl chains can insert into and destabilize the bacterial lipid membrane. However, the apparent multiple mechanisms endowing FAs with their antibacterial activity are still far from being deeply understood. Therefore, the interaction between FAs and bacterial membranes deserves to be further investigated by employing powerful surface techniques (X-ray and neutron reflectivity, infrared reflection absorption spectroscopy, among others) [3,4,5].

Among the different strategies described in the literature to transfer FAs to the bacterial membrane, FA chains were also incorporated into the liposomal bilayer, becoming essentially a component of these artificial vesicles that generally have cholesterol and other natural phospholipids as standard components of their lipid bilayer. This mechanism enables the efficient delivery of high concentrations of FAs to bacterial cell membranes, thereby enhancing their activity [6]. In fact, liposomes can fuse with bacterial membranes, facilitating the direct release of FAs into the lipid bilayer of the target cells. This interaction induces morphological alterations and increases membrane permeability, ultimately leading to bacterial cell death [7].

An alternative approach to vehicle FAs consists of encapsulating them in micelles of other surfactants. However, this strategy was less successful, since the loading capacity of micelles is lower than that of liposomes [8].

An alternative strategy for exploring the potential of FAs as antimicrobials could be the conjugation of them with suitable carriers, like hydro soluble and biodegradable compounds. In this regard, biodegradable polymers, produced from natural and renewable resources, can represent ideal candidates. We have developed several pectin samples functionalized with different FAs (palmitic, oleic, and linoleic acid) and investigated their antimicrobial activity against two foodborne pathogens, *Staphylococcus aureus* (Gram-positive) and *Escherichia coli* (Gram-negative) [9]. The results obtained have shown that, among the tested FAs, oleate and linoleate acids exhibited the greatest inhibitory effect [9].

**Figure 1:**
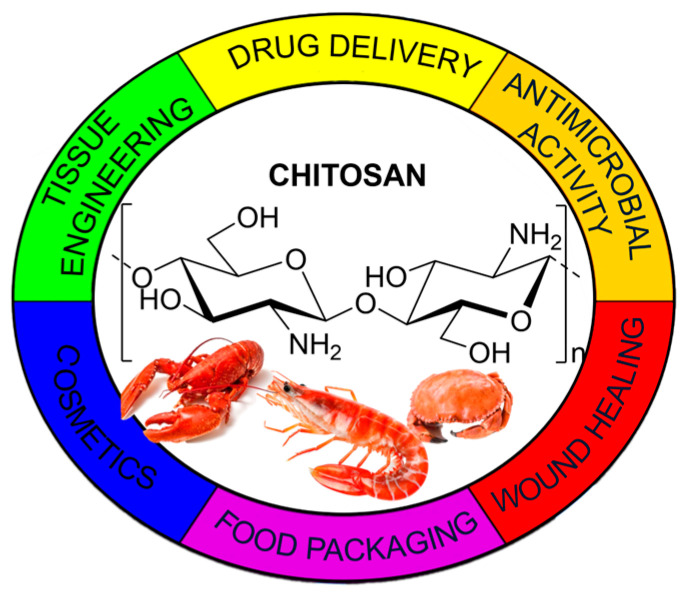
Physicochemical and biological properties of CS.

We applied a green procedure to conjugate natural FAs to the naturally occurring polysaccharide chitosan (CS), a linear cationic copolymer consisting of β-(1,4)-linked 2-acetamido-2-deoxy-β-D-glucopyranose and 2-amino-2-deoxy-β-D-glycopyranose. The conjugation protocol was a complete solvent-free procedure: the fatty acid carboxy group activation was performed in the absence of a solvent and under MW activation, as well as the subsequent amidation reaction that involves the chitosan amine group [10,11,12,13]. CS has been extensively studied for its potential in the development of drug delivery systems, owing to its biodegradability and biocompatibility (Figure 1) [14,15,16,17,18]. CS is able to form micelles upon chemical modification of its amino groups with several compounds [19,20,21,22,23,24,25,26].

Herein, we present a novel synthetic procedure for preparing CS derivatized with natural FAs. This allowed for the eco-friendly and efficient chemical modification of the polysaccharide, and the obtained conjugates were fully characterized by FT-IR and NMR spectroscopy. The self-assembling behavior of the developed CS-based conjugates was investigated, and this prompted us to explore a novel formulation for the delivery of FAs as antimicrobial agents. Specifically, the micellar aggregates formed by the CS-FA conjugates were investigated for their potential as effective delivery systems for FAs, and their antibacterial activity was evaluated against *E. coli* and *B. subtilis* [21,24].

## 2. Materials and Methods

### 2.1. Materials

CS low molecular weight (50,000-190,000 Da; deacetylation > 75%), palmitic anhydride, oleic acid, linoleic acid, DIC, K_2_CO_3_, and Mueller–Hinton (MH) were purchased from Sigma-Aldrich (St. Louis, MO, USA). Spectrum™ Labs Spectra/Por™ 3 3.5 kD MWCO Standard RC Dry Dialysis Kits were purchased from Thermo Fisher Scientific (Waltham, MA, USA); a spectrophotometer plate reader (used for absorbance measurements) was purchased from TECAN, Männedorf, Switzerland; *Escherichia coli* DH5α (used as a representative Gram-negative bacterium) was purchased from Agilent Technologies, Santa Clara, CA, USA. The laboratory strain *Bacillus subtilis* 168, subsp. *subtilis*, ATCC 23857 (used as a representative Gram-positive bacterium) was purchased from ATCC (Manassas, VA, USA).

### 2.2. Synthesis of CS–FA Conjugates

FAs (oleic and linoleic acids, 11 µL corresponding to 0.035 mmol) and N,N′-diisopropylcarbodiimide (DIC, 0.5 equiv.) were placed in a 0.5−2 mL microwave vial and mixed by using a Gilson pipet. The reaction mixture was irradiated for 2 min at 80 °C in a microwave oven (Biotage Initiator+, Sweden AB, Uppsala, Sweden). In a subsequent step, by using a glass round rod (⌀ 5.0 mm), the resulting FA anhydride and 10 mg of CS were manually milled in the same 0.5−2 mL microwave vial in the presence of ∼3 mg of K_2_CO_3_ to obtain CS−FA conjugates. The mixture was irradiated for 2 min at 80 °C in a microwave oven.

Palmitic anhydride (5 mg), commercially available, was directly manually milled with CS (10 mg) and ∼3 mg of K_2_CO_3_. The final mixture, placed in a 0.5–2 mL microwave reactor vessel, was irradiated at 160 °C for 3 min.

Then, the crude solid was dissolved in 20 mL of Milli-Q water and the pH adjusted to 7–8 by dropwise addition of 0.1 M HCl.

The solution was transferred to a 250 mL separatory funnel and extracted with ethyl acetate to remove unreacted FA.

The aqueous layer was dialyzed against Milli-Q water for 12 h using a Spectra/Por dialysis membrane with a molecular weight cut-off of 3.5 kDa. The purified product was recovered by lyophilization, yielding approximately 40%.

### 2.3. FT-IR Spectroscopy Characterization

CS-FA conjugates were characterized by FT-IR spectroscopy using the ATR accessory of the JASCO FT/IR-4100 Fourier Transform Infrared Spectrometer instrument (JASCO Europe S.r.l., Cremella, Italy). IR transmission spectra were recorded with a number of scans of 16 at a resolution of 16 cm^−1^ over the spectral range of 400−4000 cm^−1^. The relevant bands of CS and CS-FA conjugates are reported as follows.

CS: ~3359 cm^−1^ (alcoholic O-H stretching), 3297 cm^−1^ (O-H and N-H stretching), ~2915 cm-1 (C-H stretching), 1627 cm^−1^ ( C=O stretching amide II of acetyl groups), 1577 cm^−1^ (N-H bending amine I), 1376 cm^−1^ (CH_2_ bending), 1155 cm^−1^ and 1085 cm^−1^ (C-O stretching), and 898 cm^−1^ (β-glycosidic bond) in the fingerprint region.

CS-FA conjugates: ~3359 cm^−1^ (alcoholic O-H stretching), 3297 cm^−1^ (O-H and N-H stretching, 2915–2850 cm^−1^ (FA alkyl chains C-H stretching), 1672 cm^−1^ (C=O stretching amide II bond between CS-NH_2_ and FA–COOH), 1627 cm^−1^( C=O stretching amide II of acetyl groups of chitin still present in CS), 1527 cm^−1^ (N-H bending amide II), 1376 cm^−1^ (CH_2_ bending), 1155 cm^−1^ and 1085 cm^−1^ (C-O stretching), and 898 cm^−1^ (β-glycosidic bond) in the fingerprint region.

### 2.4. Preparation and Size Distribution Characterization of CS-FA Micelles (Dynamic Light Scattering)

CS-FA nanoparticles were prepared using a sonication method.

In total, 1.0 mg of CS-FA conjugate was dissolved in 1 mL of 0.9% (*w*/*v*) NaCl aqueous solution. The resulting solution was sonicated for 30 min at room temperature. After sonication, the suspension was centrifuged at 13,000 rpm for 10 min to collect the formed nanoparticles.

Dynamic light scattering (DLS) using a Zetasizer PRO instrument (Malvern Panalytical, Worcestershire, UK; Almelo, The Netherlands) was employed to determine the size distribution of the obtained nanoparticles. Measurements were conducted at 25.0 °C using disposable microcuvettes with a volume capacity of 40–45 µL.

### 2.5. Determination of Critical Micellar Concentration (CMC)

A stock pyrene solution (3.0 × 10^−2^ M in Ethanol) was diluted with Milli-Q water to achieve a final concentration of 1.2 × 10^−7^ M. Then, ethanol was eliminated via rotary evaporation at 60 °C for 1 h. The resulting aqueous pyrene solution was 4.8 × 10^−7^ M concentrated.

CS-FA conjugate (1.5 mg) was dissolved in this solution (4.8 × 10^−7^ M), and a series of dilutions were prepared to obtain the following concentrations: 1.7, 1.25, 0.62, 0.31, 0.16, 0.08, 0.04, 0.02, 0.01, 0.005, 0.002, 0.0012, 0.0006, 0.0003, and 0.00015 mg mL^−1^ of CS-FAs.

Fluorescence emission spectra were recorded using a JASCO FP-8350 ETC-115 spectrofluorometer, with excitation set at 336 nm. Emission spectra were collected in the wavelength range of 350–500 nm. The fluorescence intensity ratio of the first (I_1_ at 373 nm) to the third (I_3_ at 384 nm) vibronic peaks of pyrene was computed and plotted as a function of CS-FA concentration.

Curve fitting and graphical analysis were performed using OriginPro 2023 (OriginLab Corporation, Northampton, MA, USA).

The CMC was determined as inflection point of the sigmoidal curve obtained by plotting the I_1_/I_3_ fluorescence intensity ratio of pyrene as a function of the logarithm of CS–FA concentration.

### 2.6. Nuclear Magnetic Resonance (1H NMR) Characterization

^1^H NMR and ^1^D-DOSY liquid-state NMR spectra 180 were recorded in D_2_O at 298 K on a Bruker Avance III HD 600 MHz spectrometer 182 (Billerica, MA, USA) equipped with a cryo-probe^1^H: 600 183 MHz, acetone as internal standard at 2.22 ppm for spectra, in D_2_O 186 Bruker TopSpin 4.0.5 software. In particular, 1D-DOSY experiments were performed using a stimulated echo sequence with bipolar gradient pulses and one spoil gradient (stebpgp1s1d) with a diffusion time Δ of 300 ms and a 190 gradient duration of 1.25 ms. Samples were prepared by adding 0.6 mL of D_2_O to 1 mg of the CS-FA conjugate.

### 2.7. Antibacterial Assay

Cells from overnight cultures were harvested by centrifugation at 4 °C (1500× *g*, 30 min), resuspended in fresh Mueller–Hinton (MH) medium, and adjusted to an initial optical density (OD_600_) of 0.15, corresponding to approximately 1.0 × 10^5^ CFU per well. Experimental assays were conducted in 96-well clear, tissue culture-treated polystyrene microplates (Falcon, Männedorf, Switzerland) with a final volume of 200 µL per well. Cultures were incubated at 37 °C with continuous shaking at 80 rpm in an incubator equipped with an integrated spectrophotometric plate reader (TECAN, Männedorf, Switzerland). CS-based micelles, either alone or conjugated with FAs such as CS–palmitate, CS–oleate, and CS–linoleate, were tested at final concentrations of 5, 20, 100, and 200 µg/mL. Micelles were added at the time of bacterial inoculation. Control wells containing untreated *E. coli/B. subtilis* cultures were included in each assay. All experiments were performed in triplicate using independent biological replicates. Bacterial growth was monitored at regular intervals by measuring OD_600_ with a SPARK multimode microplate reader (TECAN, Männedorf, Switzerland). After 20 h of incubation, the OD_600_ value of the untreated control cultures was set as 100% growth. The antibacterial activity of the CS-based micelles, alone or conjugated with FAs, was expressed as the percentage of growth inhibition relative to the control.

## 3. Results and Discussion

### 3.1. Synthesis and Structural Characterization of CS–FA Conjugates

A safe and eco-friendly full solid-state protocol to conjugate FAs to CS acid was developed. The main purpose was the employment of a sustainable chemical process, from both an environmental and economic point of view, which could reduce the production of hazardous/harmful byproducts and waste [27].

Specifically, the CS-FA conjugates were prepared by a stepwise solvent-free process promoted by microwave irradiation [12,28]. It consisted of a carbodiimide-mediated amidation reaction performed as described below.

**Scheme 1:**
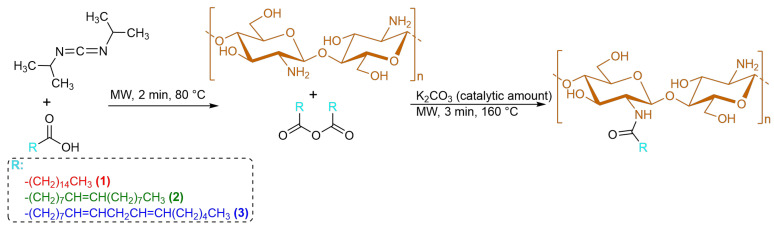
Solvent-free protocol for CS derivatives: (1) CS-palmitate, (2) CS-oleate, (3) CS-linoleate.

Oleic and linoleic acid were mixed with DIC in a microwave vial by using a pipet, both reagents being in a liquid state. The mixture was irradiated for 2 min at 80 °C in a microwave oven. In a subsequent step, CS and a catalytic amount of potassium carbonate were added to the anhydrides in order to be manually milled with the oleic, linoleic, and palmitic anhydride, which are commercially available, by using a glass round rod. The resulting solid mixture was irradiated for 3 min at 160 °C in a microwave oven (Scheme 1). Then, after cooling, the solid mixture was dissolved in water, and the unreacted FAs were removed by extracting the water layer with ethyl acetate. Subsequently, the pH of the aqueous solution was adjusted to neutrality by adding several drops of HCl aqueous solution, and residual salts were removed by dialysis.

**Figure 2:**
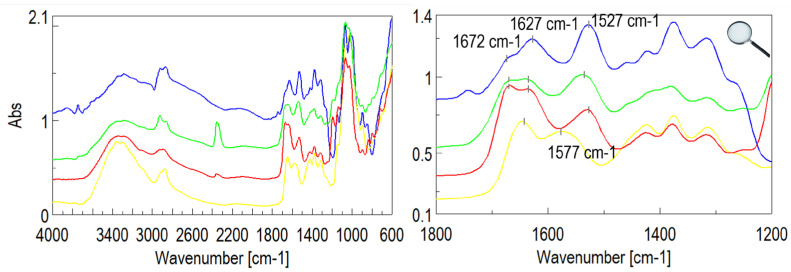
Normalized FT-IR spectroscopy of CS (yellow), CS-palmitate (red), CS-oleate (green), CS-linoleate (blue).

The successful functionalization of CS with FAs was confirmed by infrared analysis. Typical bands of CS (Figure 2) were found around 3200 cm^−1^, relative to O-H stretching; 1627 cm^−1^, taking into consideration a percentage of chitin acetyl groups still present on the CS skeleton, precisely being related to the C=O stretching amide II; 1577 cm^−1^, related to the free amine groups (N-H bending); and bands around 1000 cm^−1^ in the fingerprint region. The CS-FA conjugates, similarly to CS, showed a band relative to alcoholic O-H stretching (~3000 cm^−1^) and a band in the range of 2915–2850 cm^−1^ that appears rather increased, since this takes also into account of the FA alkyl chain C–H stretching [26,29,30].

Compared to the CS spectrum, the CS-FA conjugate spectra are characterized by a strong absorption band at 1672 cm^−1^ (this band is a shoulder for the CS-linoleate conjugate), which takes into account the new amide bond formation between CS and the FAs (C=O stretching new amide II bond). While the band at 1627 cm^−1^ is still prominent, which is related to the C=O amide II stretching of the acetyl groups, the band at 1577 cm^−1^ of the N-H bending primary amine disappears. This was replaced by a strong band at 1527 cm^−1^, which is related to the N-H bending amide II of the new formed bond. These results clearly indicated that an amide linkage between the –COOH of FA and –NH_2_ of CS is present in the conjugate CS-FAs.

**Figure 3:**
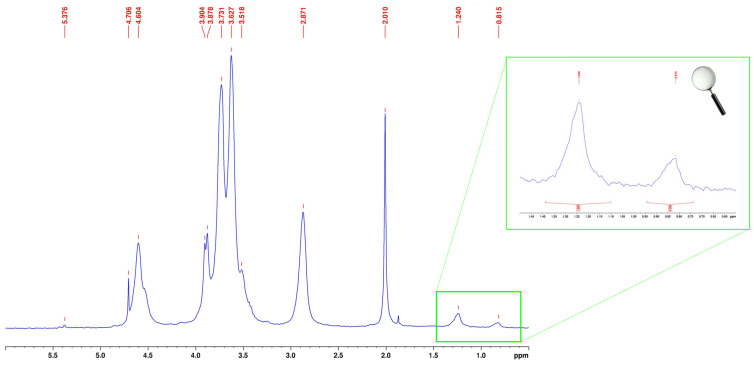
1D-DOSY NMR spectrum (400 MHz, 298 K, D_2_O) of the CS−oleate conjugate.

^1^H NMR and 1D-DOSY liquid-state NMR spectra of the CS-oleate conjugate were recorded in D_2_O and evidenced two signals at about 1.2 and 0.8 ppm due to methylene (except for those in allylic position and adjacent to the carbonyl) and methyl protons in the fatty acid moiety, respectively (see Figure S1 in Supplementary Materials). The low intensity of these signals is likely due to the amphiphilic nature of CS-FAs, which revealed a strong tendency to aggregate in micelles (see next paragraph). The hydrophobic FA chains are expected to be embedded in the inner core of the macroaggregate; however, their characteristic signals are still detectable in the D_2_O 1D-DOSY NMR spectrum (see Figure 3). In fact, the persistence of the signals at 1.2 and 0.8 ppm in 1D-DOSY NMR spectrum clearly evidenced a covalent bond between the fatty acid and the polysaccharide [10].

### 3.2. Evaluation of CMC and Investigation of CS-FA Micelles Size via Dynamic Light Scattering Technique

The obtained CS-FA conjugates were studied for their ability to self-assemble and the pyrene fluorescence method was employed [31].

Aqueous pyrene solutions containing different concentrations of CS-FA conjugates were excited at 337 nm, and an emission spectrum was analyzed by evaluating the intensity ratio at 373 nm and 384 nm (I_1_/I_3_). The analysis of this intensity ratio gives information about the environment surrounding the pyrene molecules: the I_1_/I_3_ ratio decreases with the increase in the CS-FA concentration, thus suggesting that pyrene is encapsulated within the hydrophobic interior of the aggregates formed by the polysaccharide-FA conjugate.

**Figure 4:**
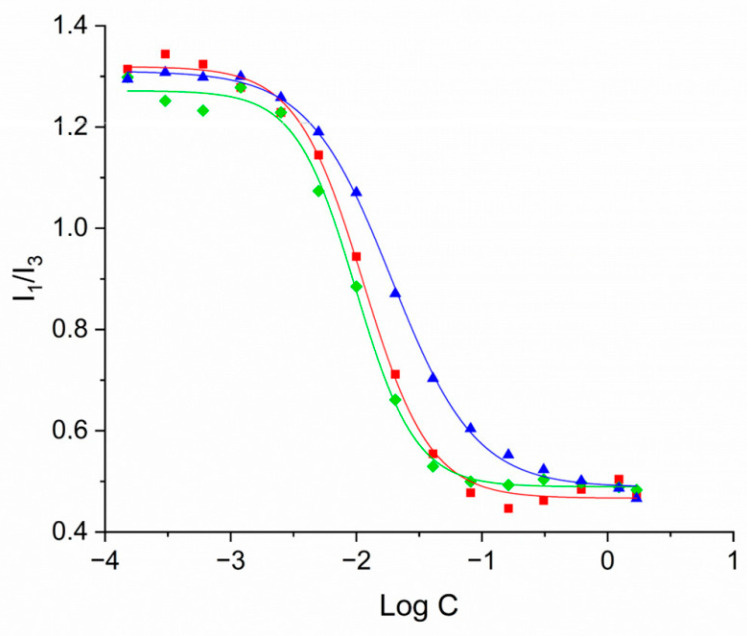
Determination of CMC by fluorescence intensity ratio (I_1_/I_3_) of CS-palmitate (red), CS-oleate (green), and CS-linoleate (blue).

The CMC was identified as an inflection point of the sigmoidal fit obtained by plotting the I_1_/I_3_ ratio versus the CS-FA concentration (Figure 4).

The CMC values obtained fall within the range of 0.0098 to 0.0191 mg/mL (Table 1), indicating the concentration range where the amphiphilic conjugate begins to form aggregates.

**Figure 5:**
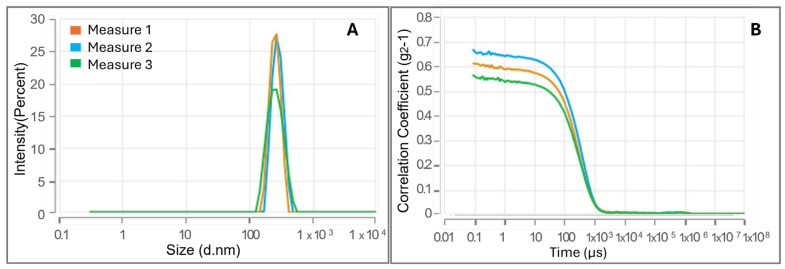
Size distribution (**panel A**) and intensity correlation functions of CS-palmitate nanoparticles (**panel B**).

**Figure 6:**
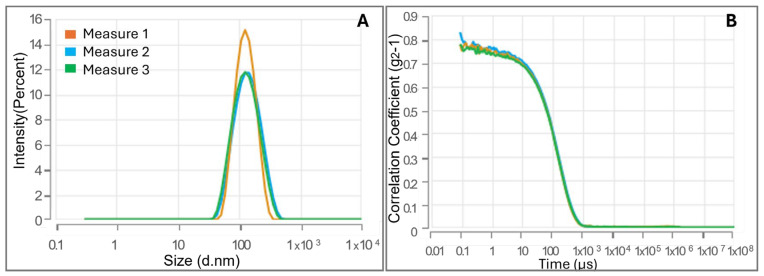
Size distribution (**panel A**) and intensity correlation functions of CS-oleate nanoparticles (**panel B**).

**Figure 7:**
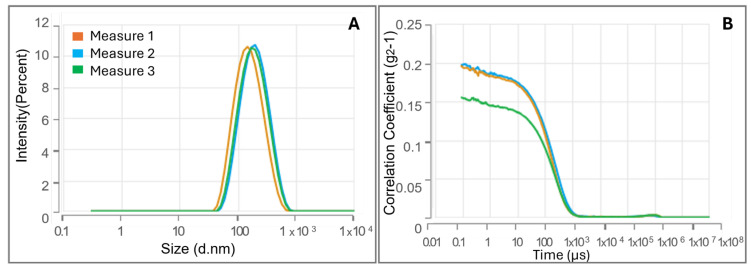
Size distribution (**panel A**) and intensity correlation functions of CS-linoleate nanoparticles (**panel B**).

Next, the nanoparticles of CS-FA conjugates were prepared by dissolving the appropriate amount of these conjugates in a 0.9% NaCl aqueous solution (see Section 2.4) and sonicating them for 30 min. Subsequently, the size distribution of the obtained aggregates was investigated with the dynamic light scattering technique (DLS). The mean diameter of the CS-FA derivatives was in the range of 109.8–252.3 nm (Figure 5, Figure 6 and Figure 7 and Table 1). A narrow size distribution was found, as indicated by the polydispersity factor which was always around 0.101–0.319. The smaller nanoparticles were formed by unsaturated FA, and the smallest and also the most soluble were revealed to be the micelles of the CS-oleate conjugate. In addition, the rather low value for the CMC of all CS-FA micelles indicated that the amphiphilic aggregates are endowed with a good water storage stability.

As expected, the functionalization of the CS amine groups increases the solubility of the polysaccharide. In fact, intra- and intermolecular hydrogen bonding between the CS molecules are reduced by the presence of acyl groups (FA chains), making the modified acylated CS more water soluble. This is not in contrast with the strong hydrophobicity of the FA chains introduced on the backbone of the polysaccharide, since the developed conjugates revealed a strong tendency to aggregate in micelles, thus directing their hydrophobic portion toward the inner part of the micelles, while the hydrophilic CS backbone is exposed to the aqueous solvent [32].

### 3.3. Antibacterial Assay Using CS-FA Micelles Against E. coli and B. subtilis

**Figure 8:**
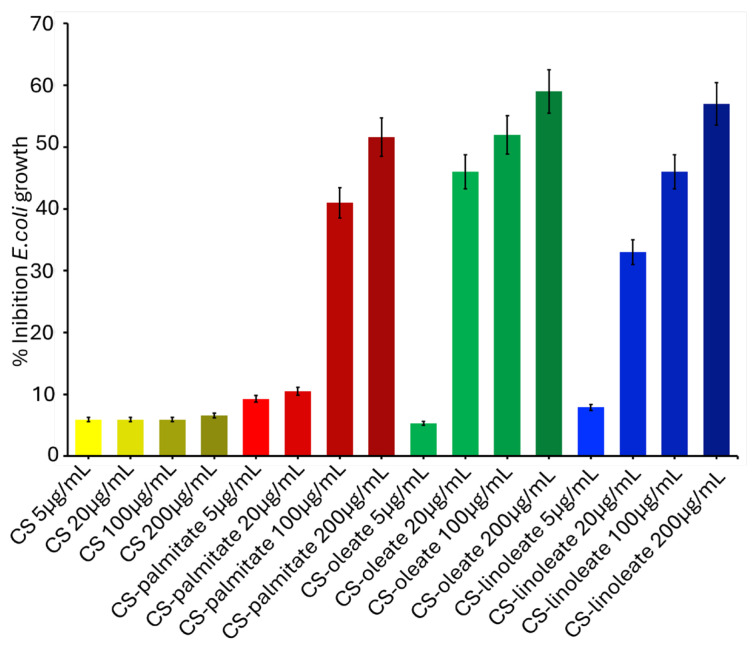
Antimicrobial activity of CS-based FA micelles against *E. coli* evaluated at various micellar concentrations.

**Figure 9:**
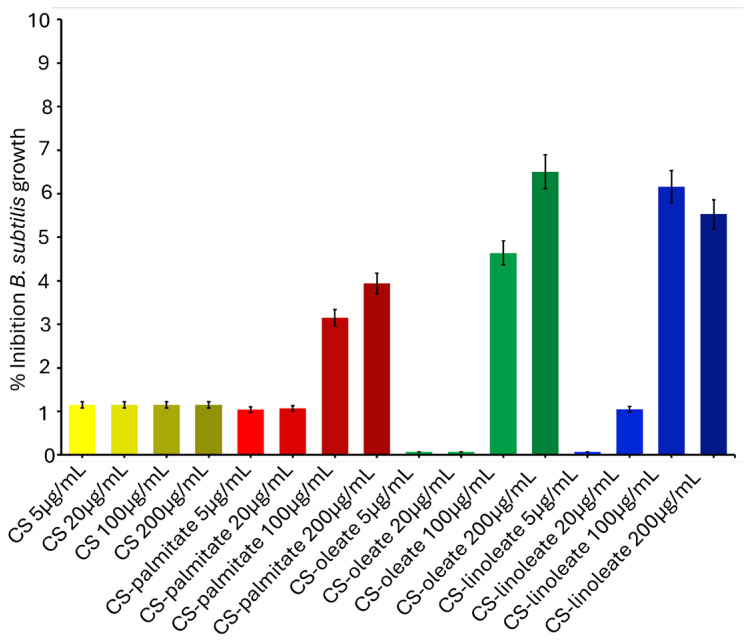
Antimicrobial activity of CS-based FA micelles against *B. subtilis* evaluated at various micellar concentrations.

The antibacterial activity of CS-FA micellar aggregates was investigated against *Escherichia coli* and *Bacillus subtilis* [33]. The bacterial growth inhibition was estimated through measurements of optical density at 600 nm and expressed as a percentage relative to untreated control cells. The antibacterial effect was observed only after fatty acid conjugation, while pristine chitosan showed no significant activity. The results are summarized in Figure 8 and Figure 9. Among the tested CS-FA conjugates, a notable inhibitory effect was observed against *E. coli*. The exposure of *E. coli* cultures to these micelles resulted in a concentration-dependent inhibition of the bacterial growth. Specifically, micelles of the conjugate CS–palmitate exhibited the most pronounced antimicrobial activity at higher concentrations, with a significant inhibition detected between 100 and 200 µg/mL. Similarly, micelles of CS–oleate and of CS–linoleate demonstrated substantial inhibitory effects across a broader concentration range, from 20 to 200 µg/mL.

Interestingly, the inhibition of *E. coli* by CS-FA micelles did not increase linearly with concentration. The saturation effect above 100 µg/mL may be explained by micelle aggregation, a limited diffusion at high concentrations, and the occupancy of accessible bacterial membrane sites. Furthermore, CS-linoleate showed a reduced activity at 200 µg/mL compared to 100 µg/mL, likely due to an excessive self-aggregation into larger assemblies, reducing effective bacterial interactions [34].

Overall, upon treatment with micellar aggregates of FA conjugated to CS, *E. coli* growth was inhibited by a percentage ranging from 35% to 60%, compared to micelles composed of pristine CS (Figure 8). This can be better understood as an indirect consequence of physicochemical interactions between the micelles and the bacterial envelope, rather than as a direct antimicrobial mechanism. Specifically, the cationic amine groups of chitosan within the micelle architecture interact electrostatically with the negatively charged lipopolysaccharides (LPS) present on the outer membrane of Gram-negative bacteria such as *E. coli*. This interaction likely perturbs membrane integrity or alters membrane-associated functions, thereby slowing bacterial proliferation without causing outright cell death.

Inhibition percentages for *B. subtilis* remained close to baseline levels, ranging from 4% to 7%, which is comparable to the effect observed with CS micelles (approximately 1% inhibition) (Figure 9). This negligible inhibitory effect observed on *Bacillus subtilis* (Gram-positive) can be mechanistically rationalized by the profound structural differences inherent to Gram-positive bacterial envelopes. *B. subtilis* features a substantially thicker peptidoglycan layer, enriched with anionic teichoic and lipoteichoic acids, which confer rigidity and serve as an effective physical and electrostatic barrier, impeding the penetration and interaction of the micelles with the underlying cytoplasmic membrane [35]. In particular, due to the extensive D-alanylation of the teichoic acids in *B. subtilis*, the net surface charge of the cell wall is significantly affected and, consequently, the electrostatic attraction to CS-based micelles might be reduced [35,36]. Additionally, the peptidoglycan matrix in *B. subtilis* exhibits a high crosslinking index and limited porosity, forming a physical barrier that may hinder the penetration of amphipathic micelles [37]. These structural features form a dynamic polyelectrolyte gel that regulates permeability and restricts the diffusion of exogenous macromolecules [38].

These findings highlight a clear differential susceptibility between the Gram-negative *E. coli* and the Gram-positive *B. subtilis* to CS-FA micelles, suggesting that the antimicrobial mechanism is likely influenced by the bacterial cell envelope composition.

It is worth underlining that the structural peculiarities of *B. subtilis* are not found for other Gram-positive pathogens, such as *Staphylococcus aureus*, which exhibits a susceptibility to CS-based micelle penetration [39]. The *S. aureus* cellular envelope is characterized by a highly anionic surface, resulting from the composition of teichoic and lipoteichoic acids with a low degree of D-alanylation [31]. In consequence of this, the *S. aureus* cell wall, while thick, is less extensively crosslinked and more permeable than that of *B. subtilis*. Furthermore, *S. aureus* lacks the biosurfactant production and stress-adaptive mechanisms found for *B. subtilis*, and its membrane composition allows for an easier insertion of FA moieties [40,41].

Another aspect to be taken into consideration concerns the chitosan pH responsiveness [42]. The antibacterial properties of the biopolymer are enhanced under acidic conditions due to an increased protonation of amino groups. Although our experiments were performed at a neutral pH, it is reasonable to hypothesize that CS-FA micelles may display an even stronger antibacterial activity in acidic environments such as infection sites. This aspect will be investigated in future studies.

## 4. Conclusions

Our studies report on a synthetic route to prepare CS-FA conjugates, following a totally solvent-free protocol. The proposed strategy, as a first step, activates the FA carboxylic functions via a carbodiimide coupling protocol, in the absence of solvent and using MW radiation to promote the reaction mixture. The subsequent amidation reaction between the obtained anhydrides and the CS was again a solvent-free process, performed in the presence of carbonate as a base and using MW irradiation. The obtained CS-FA conjugates were able to form nanomicelles that proved to be promising and effective antimicrobial agents against *E. coli*, which can be attributed to their unique structural properties and mechanisms of action, consisting of perturbing bacterial membranes. This is achieved through electrostatic interactions between the positively charged cationic groups of chitosan and the negatively charged components on the bacterial cell surface. Fatty acids can penetrate and integrate into the lipid bilayer of bacterial membranes, further compromising membrane integrity and potentially leading to cell damage and increased permeability [43]. Such interactions can destabilize the bacterial cell wall and may ultimately result in partial growth inhibition or cell lysis [38]. On the contrary, *B. subtilis* exhibits membrane structural features that hinder the diffusion of CS-FA micelles.

Our future plan is to investigate the antimicrobial activity of the developed CS-FA conjugates against other Gram-positive pathogens. The final aim is to deeply investigate the structural membrane factors that affect susceptibility to the electrostatic and amphipathic actions of CS-FA micelles.

## Figures and Tables

**Table 1:** Characteristic parameters of CS-FA micelles.

	Mean Diameter (nm)	PDI	Δ (mV)	CMC (LogC)	CMC (mg/mL)
**CS-PALMITATE**	252.3 ± 20.25	0.101 ± 0.055	17.53 ± 1.62	–1.945 ± 0.022	0.0114
**CS-OLEATE**	109.8 ± 1.972	0.239 ± 0.025	16.81 ± 1.53	–2.005 ± 0.020	0.0099
**CS-LINOLEATE**	136.2 ± 9.415	0.319 ± 0.045	17.03 ± 1.21	–1.719 ± 0.016	0.0191

## Data Availability

The original contributions presented in the study are included in the article/Supplementary Materials, further inquiries can be directed to the corresponding author.

## Supplementary Materials

The following supporting information can be downloaded at https://www.mdpi.com/article/10.3390/jfb16100373/s1. Figure S1: ^1^H NMR spectrum of CS-oleate.

## Abbreviations

The following abbreviations are used in this manuscript:

1D-DOSY	One-Dimensional Diffusion-Ordered Spectroscopy
ATCC	American Type Culture Collection
*B. subtilis*	*Bacillus subtilis*
CFU	Colony Forming Units
CMC	Critical Micellar Concentration
CS	Chitosan
CS-FA	Chitosan–Fatty Acid
D_2_O	Deuterium Oxide
DIC	N,N′-Diisopropylcarbodiimide
DLS	Dynamic Light Scattering
*E. coli*	*Escherichia coli*
FA	Fatty Acid
^1^H NMR	Proton Nuclear Magnetic Resonance Spectroscopy
HCl	Hydrochloric acid
FT-IR	Fourier Transform Infrared Spectroscopy
K_2_CO_3_	Potassium Carbonate
LPS	Lipopolysaccharides
MH	Mueller–Hinton
MW	Microwave
MWCO	Molecular Weight Cut-Off
NMR	Nuclear Magnetic Resonance
OD_600_	Optical Density at 600 nm
PDI	Polydispersity Index
*S. aureus*	*Staphylococcus aureus*
TECAN	Tecan Group Ltd.
