# Supplementary material for: Eco-Friendly Synthesis of Chitosan–Fatty Acid Nano Micelles and Their Differential Antibacterial Activity Against Escherichia coli and Bacillus subtilis
Source: J Funct Biomater. 2025 Oct 7;16(10):373. doi: 10.3390/jfb16100373 (PMC12565488; doi:10.3390/jfb16100373)
Supplement: Supplementary file 1 [file jfb-16-00373-s001.zip › jfb-3814499-supplementary.pdf]

Supporting Information

Eco-Friendly Synthesis of Chitosan–Fatty Acid Nano micelles and Their Differential Antibacterial Activity against *Escherichia coli* and *Bacillus subtilis*

Alfio Pulvirenti [1,†], Valentina Verdoliva[2,†], Viviana De Luca[3], Serena Traboni[4], Clemente Capasso[3] and Stefania De Luca [1,*]

1 Department of Biomedical Sciences, Institute of Biostructures and Bioimaging, National Research Council (CNR), Via P. Castellino, 111, 80131 Naples, Italy; alfio.pulvirenti86@gmail.com
2 Department of Environmental, Biological and Pharmaceutical Sciences and Technologies, National Research Council (CNR), Institute of Crystallography, Via Vivaldi, 43, 81100 Caserta, Italy;
valentina.verdoliva@cnr.it
3 Department of Biology, Agriculture and Food Sciences, National Research Council (CNR), Institute of Biosciences and Bioresources, Via P. Castellino, 111, 80131 Naples, Italy; viviana.deluca@ibbr.cnr.it, clemente.capasso@cnr.it
4 Department of Chemical Sciences, University of Naples Federico II, Strada Comunale Cinthia 26, 80126 Naples, Italy; serena.traboni@unina.it
* Correspondence: stefania.deluca@cnr.it (S.D.L.)
† These authors equally contributed to this work.

TABLE OF CONTENTS

- **^{1}H NMR and 1D-DOSY NMR spectrum of CS-oleate……………………..…………S2**

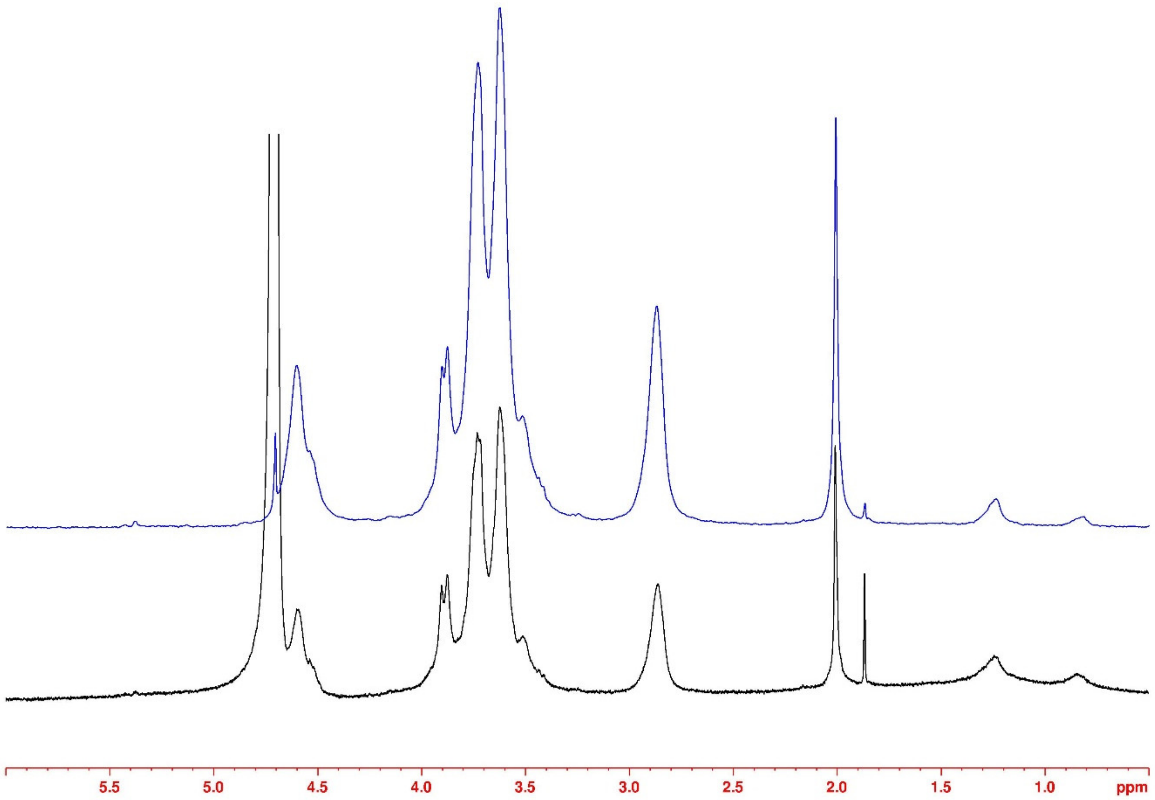

Figure S1. NMR spectra (400 MHz, 298 K, D_2O) of the CS−oleate conjugate (black: ^1H NMR; blue: 1D-DOSY NMR).
